# Analgesic and Antipyretic Activities of Ethyl Acetate Fraction Tablet of *Andrographis paniculata* in Animal Models

**DOI:** 10.1155/2021/8848797

**Published:** 2021-03-08

**Authors:** Hilkatul Ilmi, Irfan Rayi Pamungkas, Lidya Tumewu, Achmad Fuad Hafid, Aty Widyawaruyanti

**Affiliations:** ^1^Center of Natural Product Medicine Research and Development, Institute of Tropical Disease, Universitas Airlangga, Surabaya 60115, Indonesia; ^2^Undergraduate Student, Universitas Airlangga, Surabaya 60115, Indonesia; ^3^Department of Pharmaceutical Sciences, Faculty of Pharmacy, Universitas Airlangga, Surabaya 60115, Indonesia

## Abstract

**Objectives:**

To determine the analgesic and antipyretic activities of a tablet derived from *Andrographis paniculata* ethyl acetate fraction (AS201-01) in animal models.

**Methods:**

The tablet derived from AS201-01 contains an equivalent of 35 mg andrographolide per tablet. Analgesic activity was determined using an acetic acid-induced writhing test on adult male mice. A writhe was recorded by a stopwatch and was defined as the stretching of the abdomen and/or stretching of at least one hind limb. For the determination of antipyretic activity, pyrexia was induced by subcutaneous injection of 15% w/v Brewer's yeast into adult male rats. Rectal temperature was monitored at 1, 2, 3, and 4 hours after treatment.

**Results:**

The results showed that the AS201-01 tablet had analgesic and antipyretic activity. In the acetic acid-induced writhing model, AS201-01 tablet exhibited significant analgesic effect with a 66.73% reduction in writhing response at a dose of 50 mg andrographolide/kg body weight compared to the negative control group. The tablet also showed a significant antipyretic effect. The maximum antipyretic effect was observed after the third hour of administration of the AS201-01 tablet at a dose of 100 mg andrographolide/kg body weight.

**Conclusion:**

Tablet of *Andrographis paniculata* ethyl acetate fraction (AS201-01) exhibited analgesic and antipyretic activities.

## 1. Introduction

Nonsteroidal anti-inflammatory drugs (NSAID) are used worldwide to treat inflammation, pain, and fever. However, they often produce significant side effects and are toxic to various organs of the body, causing problems such as kidney failure, allergic reactions, reduced auditory ability, and increased risk of hemorrhage due to interference with platelet function [1, 2]. Therefore, the development of novel compounds with analgesic and antipyretic activities without side effects are needed [3].

Traditional uses of medicinal plants provide suitable sources for the development of new drugs [4]*. Andrographis paniculata*, commonly known as the “king of bitters”, is an herbaceous plant belonging to the Acanthaceae family. This plant is widely grown in the tropics, especially in India, Sri Lanka, Pakistan, and Indonesia [5]. *Andrographis paniculata* is one of the most popular medicinal plants used traditionally to treat fever and infectious diseases. This plant has been reported to have anti-inflammatory, antibacterial, antioxidant, anticancer, antidiabetic, antimalarial, hepatoprotective, immunostimulant, antiallergic, analgesic, and antipyretic effects [6–10].

Several studies have been conducted to determine the analgesic and antipyretic activity of *A. paniculata*. The compounds isolated from *A. paniculata*, namely, andrographolide and 14-deoxy-11,12-didehydroandrographolide, as well as the derivatives of both, showed analgesic and antipyretic activities [11]. Analgesic activity was determined *in vivo* in mice using the hot plate and writhing test, while antipyretic activity was determined *in vivo* in rats using the Baker's yeast-induced fever test. Andrographolide produced a significant analgesic effect at a dose of 4 mg/kg body weight given intraperitoneally [11–13]. Ethanol extract of *A. paniculata* showed the analgesic activity in the writhing test in mice [2]. The ethanol extract of *A. paniculata* was introduced orally in the mice, and the results showed that the *A. paniculata* ethanol extract had an analgesic activity of 34%. Sodium diclofenac, the positive control, had an analgesic activity of 76%.

Previous studies have reported that the ethyl acetate fraction of *A. paniculata* contains 27.22% higher andrographolide compared to the ethanol extract [14]. Therefore, an increased likelihood that the ethyl acetate fraction of *A. paniculata* will have stronger analgesic and antipyretic effects than the ethanol extract. However, this has never been tested. Here we show the first investigation into the analgesic and antipyretic activities of the ethyl acetate fraction tablet of *A. paniculata.*

## 2. Materials and Methods

### 2.1. Materials

The plant material used in this study was *Andrographis paniculata* powder with 1.82% andrographolide content obtained from Pharmaceutical Industry PT Kimia Farma (Persero) Tbk., Indonesia. The tablet derived from *A. paniculata* ethyl acetate fraction (AS201-01) contained an equivalent of 35 mg andrographolide per tablet and was produced at Faculty of Pharmacy, Universitas Airlangga.

### 2.2. Extraction and Fractionation

Andrographolide is the major active compound found in *A. paniculata*. It was optimally soluble in methanol and ethanol, and the solubility increased with an increase of temperature in the range of 15–50°C [15]. Ethanol can be used as an extraction solvent and safe for human consumption [16]. Therefore, the extraction was conducted using ethanol at temperature 50°C to optimize the extraction result. The *A. paniculata* powder as much as 1 kg was extracted using 6 L of 96% ethanol as a solvent. The extraction was conducted by stirring for 60 minutes at temperature of 50°C. The extract was filtered, and residue was further extracted again using 6 L of 96% ethanol. The second extract was filtered and gathered with the first extract. Total solvent used for extraction was 12 L. The collected liquid extract then evaporated to 40% of its initial volume to obtain a concentrated extract. The concentrated extract was then fractionated with water-ethyl acetate (2 : 1) mixture to obtain the ethyl acetate fraction. The liquid fraction was then evaporated to obtain a dried ethyl acetate fraction [14].

### 2.3. Determination of Andrographolide Content in Ethyl Acetate Fraction and Tablet

Andrographolide content in ethyl acetate fraction and tablet was determined by thin layer chromatography (TLC)-densitometry. Andrographolide standard (Aldrich 365645-100 MG) and samples (ethyl acetate fraction and AS201-01 tablet) were spotted on a TLC silica gel 60 GF254 plates. A chloroform-methanol solution (9 : 1) was used in the mobile phase. TLC plate was then analyzed under UV wavelength of 200–400 nm with CAMAG TLC scanner 3, and maximum absorbance was found to be 228 nm. The regression curve of the andrographolide standards was determined, and then it was used to calculate the andrographolide content in fraction and tablet [14].

### 2.4. Formulation and Production of Ethyl Acetate Fraction Tablet

The ethyl acetate fraction tablet was produced to contain 35 mg andrographolide. The amount of ethyl acetate fraction per tablet was calculated based on andrographolide content which was determined previously. Tablet composition was as follows: ethyl acetate fraction (equivalent to 35 mg andrographolide) 167.5 mg, PVP K-30 13 mg, microcrystalline cellulose (MCC) 150 mg, amylum manihot 150 mg, lactose 120 mg, PEG-4000 13.75 mg, sodium starch glycolate (SSG) 26 mg, talk 4.875 mg, and Mg stearate 4.875 mg. The total weight of the tablet was 650 mg.

Tablets were produced on the laboratory scale, 100 tablets per batch. To produce the tablet, first, the ethyl acetate fraction was dissolved in a sufficient quantity of ethanol. PEG-4000 and PVP K-30 were then added and mixed. MCC was then added as a diluent, followed by amylum manihot and lactose. The mixture was then dried at temperature 40°C for 12 hours and sifted through an 18-mesh sieve (1 mm). SSG, talk, and Mg stearate were added as a diluent and lubricant, respectively, and then mixed well for 15 min. The prepared mixture was compressed into a tablet using a 13 mm punch on a tablet machine.

### 2.5. Evaluation of Tablet

The tablet of *A. paniculata* ethyl acetate fraction (AS201-01) was evaluated for following parameters [17].

### 2.6. Tablet Weight Variation

The high weight variation of the tablet had the ability to influence the dose. Therefore, a weight variation test was carried out. Twenty tablets were weighed individually using an electronic balance (Precisa), and the average weight was calculated.

### 2.7. Hardness

Tablets must have a certain hardness to withstand mechanical shocks. Tablet hardness can also affect the dissolution release, influencing the bioavailability of the drug. Therefore, a hardness test was conducted using a hardness tester (Schleuniger). A runway driven by an electric motor pressed the tablet until the tablet breaks, a scale instruction gives the breaking strength value (kg/cm^2^). The recommended value is 4–8 kg/cm^2^ which indicates hardness of tablets.

### 2.8. Friability

The tablets are weighed, and placed on a device (Friabilator) then operate device as much as 100 revolutions (25 rpm). Then, the tablets were dedusted and reweighed. Weight loss should not exceed 0.5–1%. Percent friability (%*F*) was calculated as follows:

(1) $$\begin{array}{c} \text{\%}F=\;\frac{\text{loss in weight}}{\text{initial weight }}\text{ x }100. \end{array}$$

### 2.9. Disintegration Time

Disintegration time was assessed using disintegration apparatus at 37 ± 2°C. We placed 1 tablet in each of the 6 tubes of basket-rack assembly, and the apparatus was operate using water. We observed the time needed for tablets to disintegrate completely. After 30 minutes, the basket-rack assembly from fluid was lifted, and we observed the tablets. All of the tablets should have disintegrated completely. If 1 or 2 tablets fail to disintegrate, we repeated the test on 12 additional tablets. No less than 16 of 18 tested tablets must be completely disintegrated after 30 minutes.

### 2.10. Experimental Animal

Male mice (BALB/C strain, 25–30 g) were used for the analgesics activity test, and male rats (Wistar strain, 100–150 g) were used for the antipyretics activity test. The animals were maintained on a standard animal pellets diet (NUVO pellets) and water *ad libitum* at the Animal Laboratory of the Institute of Tropical Disease, Universitas Airlangga, Surabaya. The animals were kept at standard temperature (25 ± 1°C) and a 12/12 h light/dark cycle. All the animals were acclimatized for seven days before the study [4]. Permission and approval for animal studies were obtained from the Faculty of Veterinary Medicine, Universitas Airlangga, with approval number 753-KE.

### 2.11. Analgesic Activity Test Using Acetic Acid-Induced Writhing Test

The analgesic activity was determined by the acetic acid abdominal constriction test [18, 19]. Twenty-five male mice were randomly divided into 5 groups, where each group consisted of 5 mice. Group 1 was treated with carboxymethyl cellulose (CMC-Na 0.5%) (as negative control). Group 2 was treated with standard drug diclofenac sodium at a dose of 40 mg/kg body weight [2]. Groups 3, 4, and 5 were treated with AS201-01 tablets at a dose equal to 12.5, 25, and 50 mg andrographolide/kg body weight, respectively. All treatments were administered orally. Thirty minutes after administration of all treatments, each mouse was injected with 1% acetic acid at a dose of 10 ml/kg body weight intraperitoneally [20, 21]. At 5, 15, 25, 35, and 45 minutes after acetic acid injection, the number of writhing responses observed during a 5-minute period were counted and recorded [11].

The percentage of analgesic activity was calculated as follows:

(2) $$\begin{array}{c} \text{\% inhibition }=\frac{\text{Wc}-\text{Wt}}{\text{Wc}}\text{ x }100\text{\%,} \end{array}$$

where *W* is the number of writhing, *c* is the negative control, and *t* is the test.

### 2.12. Antipyretic Activity Test Using Yeast-Induced Hyperthermia in Rats

The antipyretic activity was evaluated with a fever induced by Brewer's yeast (Sigma 51475) following the established method in rats with some modifications [13]. The normal temperature was recorded before injection of Brewer's yeast using the rectal route using a digital probe thermometer for rats (BIOSEP®) to a depth of 3 cm into the rectum. Pyrexia was induced by subcutaneous injection of 20% w/v suspension of Brewer's yeast in distilled water at a dose of 10 mg/kg body weight. After 18 hours, the rise in rectal temperature was recorded, and only animals showing an increase in temperature of at least 0.6°C were selected for the study. The animals were randomly divided into six groups, each group containing five rats. Group 1 was treated with CMC-Na 0.5% (as negative control). Group 2 was treated with standard drug paracetamol at a dose of 150 mg/kg body weight [21]. Groups 3, 4, 5, and 6 were treated with AS201-01 tablets at a dose equal to 12.5, 25, 50, and 100 mg andrographolide/kg body weight, respectively. All treatments were administered orally. After the treatments, the rectal temperature of each animal was again recorded at 1 hour intervals up to 4 hours. The percentage reduction in pyrexia was calculated using the following formula:

(3) $$\begin{array}{c} \text{\% reduction}=\frac{B-\mathrm{Cn}}{B-A}\times 100\text{\%,} \end{array}$$

where *A* is the normal temperature, *B* is the rectal temperature after 18 h of yeast injection, and *Cn* is the rectal temperature after 1, 2, 3, and 4 h.

### 2.13. Data Analysis

The results obtained were expressed as the mean ± SEM (standard error of mean) of six animals. For statistical analysis, one-way ANOVA was followed by post-hoc Dunnett's test for multiple comparisons. An effect was considered to be significant at the *P* < 0.05 level. GraphPad Prism 7.0 software (GraphPad Co., Ltd., San Diego, CA, USA) was used in statistical analysis.

## 3. Results

### 3.1. Determination of Andrographolide Content in Ethyl Acetate Fraction and Tablet

Andrographolide content in ethyl acetate fraction was determined by the TLC-densitometry method using andrographolide (Aldrich 365645-100 MG) as a standard. Andrographolide content in the pure ethyl acetate fraction was 20.90 ± 2.72% and 6.51 ± 0.20% in the AS201-01 tablet manufactured from this fraction (Tables 1 and 2). This result was used when preparing doses of 12.5 mg andrographolide/kg body weight.

### 3.2. Formulation, Production, and Evaluation of Ethyl Acetate Fraction Tablet

Several physical characteristics of the AS201-01 tablet were assessed according to the method of Depkes RI, 2014 [17]. The AS201-01 tablet has specifications which are shown in Table 3. The tablet meets the specification requirements by Farmakope Indonesia.

### 3.3. Analgesic Activity

The effect of AS201-01 tablet on acetic acid-induced writhing in mice is presented in Table 4. The results indicated that AS201-01 tablet significantly reduced (*P* < 0.0001) in the writhes count after oral administration in a dose-dependent manner when compared to the negative control. The maximum inhibition was observed at 50 mg andrographolide/kg dose of AS201-01 tablet (66.73%). However, diclofenac sodium (reference drug) reduced the number of abdominal writhes by 74.62%. Statistical analysis showed a significant difference in the three doses compared to diclofenac sodium (*P* < 0.05). The inhibitory effect of diclofenac sodium was greater than that of the highest dose of the AS201-01 tablet.

### 3.4. Antipyretic Activity

The effect of AS201-01 tablet and paracetamol in yeast-induced pyrexia in rats is shown in Table 5. The subcutaneous injection of yeast increased the rectal temperature by 1.26–2°C after 18 hours of injection.

**Figure 1:**
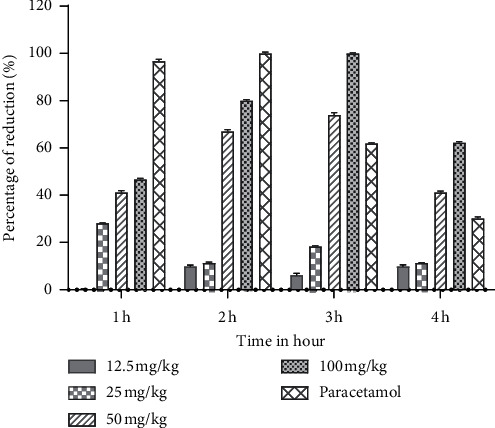
Percentage reduction of AS201-01 tablet and standard drug (paracetamol) in yeast-induced pyrexia in rats.

The AS201-01 exhibited an antipyretic effect during the first hour after administration in a dose-dependent manner which was significantly different (*P* < 0.05) from the negative control. The dose of 50 and 100 mg/kg body weight significantly attenuated pyrexia in rats after 1 hour (*P* < 0.05), and the lowering of temperature was even more significant (*P* < 0.001) from 2 h to 3 h in comparison to the negative control. The maximum reduction was observed at 100 mg andrographolide/kg dose of AS201-01 tablet after the third hour of administration (100%). The data are shown in Figure 1. Meanwhile, the maximum reduction was showed by paracetamol (reference drug) after the second hour of administration (100%). The percentage of reduction was decreased during the third hour and showed the lowest after the fourth hour of paracetamol administration. This activity profile was probably attributed to their pharmacokinetics characteristics.

## 4. Discussion

Medicinal plants are important sources for the development of new drugs because most of these products are believed to have bioactive compounds responsible for healing various diseases without any side effects and at a lower cost [22]. *Andrographis paniculata* is one of the most popular medicinal plants used traditionally and known to exhibit a wide range of pharmacological effects. Andrographolide is a major constituent of *A. paniculata* and is likely to be responsible for the analgesic and antipyretic effect of *A. paniculata* [11]. Madav et al. have reported that 300 mg/kg of andrographolide, administered orally, had a significant analgesic activity on acetic-induced writhing in mice at doses of 100 and 300 mg/kg body weight [12]. In addition, doses of 180 and 360 mg/kg body weight of andrographolide were also found to be able to relieve fever in humans by the third day after administration [23].

*A. paniculata* is widely used in traditional medicine and safely consumed. Oral acute toxicity evaluation reported an ethanolic extract of *A. paniculata* with an upper fixed dose of 5000 mg/kg body weight, which has no significant acute toxicological effects [24]. Meanwhile, andrographolide as a bioactive compound of *A. paniculata* was reported to have LD_50_ higher than 5 g/kg body weight by oral treatment both in male and female mice [25]. These data support the safe use of *A. paniculata* as alternative medicine.

Previous studies have reported that the ethyl acetate fraction of *A. paniculata* has a higher andrographolide content than the ethanol extract [14]. Based on the results, ethyl acetate fraction was further developed as a tablet dosage form, namely, AS201-01 tablet. The determination of andrographolide content in ethyl acetate fraction was conducted by the TLC-densitometry method. The data were used to manufacture AS201-01 tablet using ethyl acetate fraction as an active ingredient which is equal to 35 mg of andrographolide per tablet. Ethyl acetate fraction (167.5 mg) which was containing 35 mg andrographolide, was needed to produce one tablet. Ethyl acetate fraction possibly contains other diterpenoids, flavonoids, and polyphenol compounds. *A. paniculata *is known as a source of 2′-oxygenated flavonoids and labdane type diterpenoids [26–28]. This study used andrographolide as the active marker of *A. paniculata* due to its abundance and various bioactive properties. Therefore, the characterization of other compounds contained in ethyl acetate was not performed.

AS201-01 tablet was then investigated for its analgesic and antipyretic activities. Analgesic activity was determined using an acetic acid-induced writhing test. Acetic acid-induced writhing reflex model in mice is a widely accepted, simple, sensitive, and effective pain model for evaluating peripherally acting analgesics [29, 30]. The characteristic of pain activity generated by intraperitoneal injection of acetic acid is presented with contraction of the abdominal muscle followed by extension of hind limbs and elongation of body parts, and such constriction is thought to be mediated by the local peritoneal receptor [31]. Acetic acid induces inflammatory pain by impelling capillary permeability [32] and releasing substances that excite pain nerve endings such as serotonin, histamine, bradykinins, and prostaglandins (PGE2 and PG2*α*) from arachidonic acid through cyclooxygenase (COX) enzymes [33, 34]. When prostaglandin is released, the nerve endings respond to it through prostaglandin *E*2 (PGE2) receptor by picking up and transmitting the pain and injury messages through the nervous system to the brain and cause visceral writhing stimuli in mice. The inhibition of prostaglandin synthesis is remarkably efficient as an antinociceptive mechanism in visceral pain [35, 36]. Diclofenac sodium was used as the positive control. It is a nonsteroidal anti-inflammatory drug (NSAID) that has analgesic, antipyretic, and anti-inflammatory activity. Diclofenac performs its action via inhibition of prostaglandin synthesis by inhibiting COX-1 and COX-2[37]. In our study, the *A. paniculata* tablet significantly reduced the number of writhing in a dose-dependent manner. The maximum inhibition was observed at 50 mg andrographolide/kg dose of AS201-01 tablet (66.73%). These findings strongly suggest that AS201-01 tablet showed its analgesic activity through a peripheral mechanism, which is the inhibition of prostaglandin biosynthesis by acting on visceral receptors sensitive to acetic acid [38].

The present study was also conducted to evaluate the antipyretic activity of the AS201-01 tablet on animal models (rats). The baker's yeast-induced fever test, which simulates a pathogenic fever, is a low-cost and reliable method for assessing new antipyretics [13, 39]. Fever is known to be caused by several endogenous pyrogens such as interleukin-1*β* (IL-1*β*) and IL-6, interferon-*α* (IFN-*α*), tumor necrosis factor-*α* (TNF-*α*), macrophage protein-1, and prostaglandins such as PGE2 and PGI2. Brewer's yeast induces both TNF-*α* and prostaglandin synthesis [40, 41].

The oral administration of the AS201-01 tablet significantly attenuated rectal temperature of yeast-induced pyrexia in rats. The efficacy of the antipyretic effect of the AS201-01 tablet was observed to have increased in a dose-dependent manner. The maximum reduction was observed at 100 mg andrographolide/kg dose of the AS201-01 tablet after the third hour of administration (100%). The inhibition of prostaglandin synthesis and the inhibition of cytokine release could be the possible mechanism of antipyretic actions of AS201-01 tablet. Andrographolide is the major compound of *A. paniculata* was reported to have analgesic, antipyretic, and anti-inflammatory activity, but the exact mechanism remains unknown. Shen et al. reported that the anti-inflammatory effect of andrographolide was explained by its ability to inhibit neutrophil adhesion/transmigration through suppression of Mac-1 upregulation [42].

Paracetamol was used as a positive control in this study. It is a standard drug with a central analgesic effect and is due to activation of descending serotonergic pathway [43]. The maximum reduction of pyrexia was showed by paracetamol as a reference drug after the second hour of administration (100%). Paracetamol is extensively metabolized and excreted unchanged in the urine, only 2–5% of its therapeutic dose. It is rapidly and relatively uniformly distributed in the tissues, and the plasma half-life is 1.5–2 hours [44]. The rapid absorption of paracetamol resulted in a high percentage of reduction pyrexia in the first hour after drug administration and reached the maximum reduction after the second hour of administration. The activity of paracetamol was decreased during the third hour and showed the lowest activity after the fourth hour of administration. The activity profile of paracetamol was in accordance with its pharmacokinetics characteristics. On the other hand, andrographolide takes a longer time to reach maximum reduction compared to paracetamol. The pharmacokinetics and oral bioavailability of andrographolide in rats and humans were studied by Panossian et al. The study reported that the maximum concentration of andrographolide in rat's plasma is estimated at 2 hours after administration, and the plasma half-life is 3 hours. Furthermore, a large part (55%) of andrographolide is bound to plasma proteins, and only a limited amount can enter the cells [45]. The Biopharmaceutics Classification System (BCS) classified andrographolide as class III drug which has low solubility and low permeability [46]. It showed poor oral bioavailability due to its high lipophilicity, low aqueous solubility rapid transformation, and efflux by P-glycoprotein [47]. This andrographolide pharmacokinetics profile indicated that AS201-01 tablet possibly needed more time to produce the maximum antipyretic activity compared to paracetamol. AS201-01 tablet still showed higher activity during the fourth administration compared to paracetamol. It was suggested that the AS201-01 tablet was potential an antipyretic drug.

Various studies reported the analgesic and antipyretic activity of *A. paniculata* extract or its compounds. On the other hand, little attention has been directed toward the development of *A. paniculata* as a herbal medicine product. This study demonstrated the analgesic and antipyretic activity of formulated ethyl acetate fraction of *A. paniculata* in the tablet dosage form. The formulation study of ethyl acetate fraction as a dosage form needed to be further conducted specially to enhance the bioavailability and reduce time to achieve maximum concentration in the plasma.

## 5. Conclusions

The ethyl acetate fraction tablet of *A. paniculata* exhibited analgesic and antipyretic activities. The maximum analgesic and antipyretic activity was observed at 50 mg andrographolide/kg and 100 mg andrographolide/kg dose of ethyl acetate fraction tablet of *A. paniculata*.

## Figures and Tables

**Table 1:** Andrographolide content in ethyl acetate fraction.

Andrographolide standard		Ethyl acetate fraction		
Concentration, *μ*g (*x*)	Area (*y*)	Area	Concentration (% w/w)	Average concentration (% w/w)^*∗*^
0.08	1042.45	3000.27	23.48	20.90 ± 2.72
0.1	1196.10	2399.68	18.05	
0.2	2531.88	2778.27	21.15	
0.3	3171.55			
0.4	4046.33			
0.6	5954.67			

Regression: *y* = 9327.3*x* + 378.86; ^*∗*^data represent mean ± SD.

**Table 2:** Andrographolide content in AS201-01 tablet.

Andrographolide standard		AS201-01 tablet		
Concentration, *μ*g (*x*)	Area (*y*)	Area	Concentration (% w/w)	Average concentration (% w/w)^*∗*^
0.08	912.82	3098.30	6.31	6.51 ± 0.20
0.1	1204.85	3171.69	6.51	
0.2	2223.54	3259.70	6.71	
0.3	3152.31			
0.4	3989.96			
0.6	5591.52			

Regression: *y* = 8948*x* + 340.39; ^*∗*^data represent mean ± SD.

**Table 3:** Physical characteristics of AS201-01 tablet.

Tablet weight (mg)				Hardness (kg/cm^2^)	Friability (%)	Disintegration time
643.2	649.4	646.6	642.2	6.26	0.80	17 min 28 sec
647.8	645.5	643.8	642.6	6.23	0.80	15 min 29 sec
645.3	643.1	643.5	642.8	6.60	0.70	
647.0	645.9	644.8	648.5			
645.8	649.1	642.1	648.6			
Average 645.38 ± 0.2				6.36 ± 0.21	0.77 ± 0.06	16 min 28.5 sec

**Table 4:** Analgesic activity of AS201-01 tablet by acetic acid-induced writhing in mice.

Group	Dose (mg/kg)	Number of writhes in 45 min (Mean ± SEM)	Inhibition (%)
Negative control	—	106.4 ± 1.1	—
Positive control	40	27 ± 1.9^*∗∗∗∗*^	74.62
AS201-01 tablet	12.5	81 ± 1.0^*∗∗∗∗*^	23.87
	25	47.2 ± 2.1^*∗∗∗∗*^	55.64
	50	35.4 ± 1.2^*∗∗∗∗*^	66.73

Data are reported as mean ± SEM for all groups. The data were analyzed by ANOVA followed by Dunnett's test. Asterisks (^*∗*^) indicate statistically significant value from negative control, ^*∗∗∗∗*^*P* < 0.0001.

**Table 5:** Antipyretic activity of AS201-01 tablet in yeast-induced pyrexia in rats.

Group (mg/kg)	Rectal temperature (°C)					
	Normal	Initial temperature (after 18 h)	After treatment			
			1 h	2 h	3 h	4 h
Negative control	36.48 ± 0.28	38.48 ± 0.22	38.16 ± 0.19	38.18 ± 0.19	38.32 ± 0.15	38.36 ± 0.19
AS201-01 tablet at dose 12.5	36.16 ± 0.14	37.78 ± 0.15	38.14 ± 0.19	37.62 ± 0.22	37.68 ± 0.32	37.62 ± 0.33
AS201-01 tablet at dose 25	36.20 ± 0.09	37.62 ± 0.21	37.22 ± 0.08^*∗*^	37.46 ± 0.17^*∗*^	37.36 ± 0.12^*∗*^	37.46 ± 0.07^*∗*^
AS201-01 tablet at dose 50	36.36 ± 0.09	38.06 ± 0.15	37.36 ± 0.31^*∗*^	36.92 ± 0.31^*∗*^	36.80 ± 0.39^*∗∗∗*^	37.36 ± 0.24^*∗*^
AS201-01 tablet at dose 100	36.30 ± 0.19	38.10 ± 0.10	37.26 ± 0.18^*∗*^	36.66 ± 0.17^*∗∗*^	36.22 ± 0.10^*∗∗∗*^	36.98 ± 0.24^*∗∗*^
Paracetamol at dose 150	36.68 ± 0.13	37.94 ± 0.21	36.72 ± 0.31^*∗∗∗*^	36.64 ± 0.27^*∗∗*^	37.16 ± 0.14^*∗∗*^	37.56 ± 0.25

Data are reported as mean ± SEM (*n* = 5). The data were analyzed by ANOVA followed by Dunnett's test. Asterisks (^*∗*^) indicate statistically significant value compared to the negative control, ^*∗*^*P* < 0.05; ^*∗∗*^*P* < 0.01; and ^*∗∗∗*^*P* < 0.001.

## Data Availability

The data used to support the findings of this study are included within the article.
